# Early high flow nasal cannula therapy in bronchiolitis, a prospective randomised control trial (protocol): A Paediatric Acute Respiratory Intervention Study (PARIS)

**DOI:** 10.1186/s12887-015-0501-x

**Published:** 2015-11-14

**Authors:** Donna Franklin, Stuart Dalziel, Luregn J. Schlapbach, Franz E. Babl, Ed Oakley, Simon S. Craig, Jeremy S. Furyk, Jocelyn Neutze, Kam Sinn, Jennifer A. Whitty, Kristen Gibbons, John Fraser, Andreas Schibler, on behalf of PARIS and PREDICT

**Affiliations:** Paediatric Critical Care Research Group, Lady Cilento Children’s Hospital and The University of Queensland, Brisbane, Australia; The University of Queensland, School of Medicine, Brisbane, Australia; Mater Research Institution The University of Queensland, Brisbane, Australia; Starship Children’s Hospital, Auckland, New Zealand; Liggins Institute, University of Auckland, Auckland, New Zealand; Department of Pediatrics, Inselspital, University of Bern, Bern, Switzerland; Emergency Department, Royal Children’s Hospital, Melbourne, Australia; Murdoch Children’s Research Institute Melbourne, Melbourne, Australia; University of Melbourne, Melbourne, Australia; Emergency Department, Monash Children’s Hospital, Melbourne, Australia; Monash University, Melbourne, Australia; Emergency Department, The Townsville Hospital, Townsville, Australia; James Cook University, Townsville, Australia; KidzFirst Middlemore Hospital, Auckland, New Zealand; University of Auckland, Auckland, New Zealand; Emergency Department, The Canberra Hospital, Canberra, Australia; Australian National University, Canberra, Australia; Paediatric Research in Emergency Departments International Collaborative (PREDICT), Brisbane, Australia; School of Pharmacy, Faculty of Health and Behavioural Sciences, The University of Queensland, Brisbane, Australia; Critical Care Research Group, The Prince Charles Hospital, Brisbane, Australia; Paediatric Intensive Care Unit, Paediatric Critical Care Research Group (PCCRG), Lady Cilento Children’s Hospital and The University of Queensland, 501 Stanley St, South, Brisbane, Queensland 4101 Australia

## Abstract

**Background:**

Bronchiolitis imposes the largest health care burden on non-elective paediatric hospital admissions worldwide, with up to 15 % of cases requiring admission to intensive care. A number of previous studies have failed to show benefit of pharmaceutical treatment in respect to length of stay, reduction in PICU admission rates or intubation frequency. The early use of non-invasive respiratory support devices in less intensive scenarios to facilitate earlier respiratory support may have an impact on outcome by avoiding progression of the disease process. High Flow Nasal Cannula (HFNC) therapy has emerged as a new method to provide humidified air flow to deliver a non-invasive form of positive pressure support with titratable oxygen fraction. There is a lack of high-grade evidence on use of HFNC therapy in bronchiolitis.

**Methods/Design:**

Prospective multi-centre randomised trial comparing standard treatment (standard subnasal oxygen) and High Flow Nasal Cannula therapy in infants with bronchiolitis admitted to 17 hospitals emergency departments and wards in Australia and New Zealand, including 12 non-tertiary regional/metropolitan and 5 tertiary centres. The primary outcome is treatment failure; defined as meeting three out of four pre-specified failure criteria requiring escalation of treatment or higher level of care; i) heart rate remains unchanged or increased compared to admission/enrolment observations, ii) respiratory rate remains unchanged or increased compared to admission/enrolment observations, iii) oxygen requirement in HFNC therapy arm exceeds FiO_2_ ≥ 40 % to maintain SpO_2_ ≥ 92 % (or ≥94 %) or oxygen requirement in standard subnasal oxygen therapy arm exceeds >2L/min to maintain SpO_2_ ≥ 92 % (or ≥94 %), and iv) hospital internal Early Warning Tool calls for medical review and escalation of care. Secondary outcomes include transfer to tertiary institution, admission to intensive care, length of stay, length of oxygen treatment, need for non-invasive/invasive ventilation, intubation, adverse events, and cost.

**Discussion:**

This large multicenter randomised trial will allow the definitive assessment of the efficacy of HFNC therapy as compared to standard subnasal oxygen in the treatment of bronchiolitis.

**Trial registration:**

The trial is registered with the Australian and New Zealand Clinical Trials Registry ACTRN12613000388718 (registered on 10 April 2013).

## Background

Bronchiolitis, an acute lower airway lung disease caused by respiratory viruses, is the most common reason worldwide for infants <1 year of age to be admitted to hospital. Data from the USA shows that the health care burden from infants hospitalised with bronchiolitis is conservatively estimated to be US$1.73 billion per year [1, 2]. In Australia, approximately 11,000 infants with bronchiolitis require hospital admission each year, of which 1254 (12 %) were admitted to a Paediatric Intensive Care Unit (PICU) in 2013. This equates to 17.1 % of all non-elective PICU admissions, imposing a heavy resource burden (http://www.anzics.com.au/Pages/CORE/CORE-Reports.aspx). Numerous studies over the last three decades have investigated the role of various medications in managing infants with bronchiolitis including adrenaline, steroids, salbutamol/albuterol, and hypertonic saline; none of these studies have definitively changed the outcome of the disease, the burden on health care systems, or reduced the number of PICU admissions [3].

The agreed current approach to management of infants with bronchiolitis in hospitals is focused on oxygen therapy for hypoxia, respiratory support and the maintenance of hydration [4]. Respiratory support has traditionally been the domain of intensive care settings, and has been provided through an escalation of therapy from simple oxygen delivery by nasal cannula, to non-invasive ventilation with Continuous Positive Airway Pressure (CPAP) and finally to intubation and mechanical ventilation [5–7]. These latter two strategies require highly skilled staff, are costly, and are associated with a greater incidence of adverse events including ventilator-induced lung injury, barotrauma, and potential neurotoxicity associated with sedation [8].

Over the last decade High Flow Nasal Cannula (HFNC) therapy has emerged as a new method to provide respiratory support for bronchiolitis [9]. HFNC therapy works by delivering an increased volume of air and oxygen into the nasal passages than standard subnasal oxygen therapy, using a higher flow of humidified and heated gas. These increased flow rates exceed peak inspiratory flow and thereby result in more efficient delivery of oxygen to the terminal airways. Physiological studies have demonstrated reduced work of breathing and improved gas exchange [10, 11]. However, the clinical effectiveness of HFNC therapy in bronchiolitis has only been reported in non-experimental observational studies, and there is a lack of appropriately powered randomised studies with well-defined clinically meaningful outcomes [12, 13]. Moreover, the potential impact of HFNC therapy on healthcare costs related to the management of infants with bronchiolitis has not been investigated.

Two previous clinical studies using HFNC therapy in a non-randomised design have shown a reduction in intubation rates in critically ill infants in the intensive care setting [14, 15]. Both studies, however, leave the question unanswered whether this is due to improved patient care or indeed to a higher admission rate of less sick patients. A recent retrospective study showed that HFNC therapy is similar in effectiveness to nasal CPAP when used in a Paediatric Intensive Care Unit (PICU) with 25 % of the patients requiring escalation to invasive ventilation [16]. Another study analysed the outcome of infants and children presenting to an emergency department (ED) including a range of diagnoses for respiratory distress [17]. Approximately half of the 498 patients studied presented with bronchiolitis. Infants with bronchiolitis were the least likely to require intubation in this cohort and most patients responded to HFNC therapy within the first three hours of HFNC therapy initiation.

Published reports on the use of HFNC therapy suggest a very good safety profile, including the fact that patients rarely require sedation in contrast to mask-delivered non-invasive ventilation [16]. Pilot studies have thus investigated use of HFNC therapy in general paediatric ward settings with encouraging results. A pilot study in 61 infants with bronchiolitis using a flow rate of 2 L/kg/min showed that HFNC therapy can be safely delivered in a general paediatric ward and that the PICU admission rate compared to a control group could be significantly reduced by 2.5 times [12]. This data suggests a beneficial role for HFNC therapy, and indicates that this can be safely implemented in ward settings. However, in view of the lack of high-grade evidence, there is an urgent need to perform a large multicentre randomised controlled trial on use of HFNC in bronchiolitis.

## Methods

### Study aims

To perform a randomised controlled multicentre trial (RCT) in infants aged less than 12 months (corrected age) with clinically diagnosed bronchiolitis comparing HFNC therapy to standard subnasal oxygen therapy. The primary outcome is treatment failure of HFNC therapy or standard subnasal oxygen.

Secondary outcome measures comprise (a) the rate of transfer of children to higher level care (that is, the rate of transfer from regional hospitals to tertiary centres and rate of transfer from tertiary hospitals to their onsite intensive care unit (ICU) if the primary admission is to their tertiary hospital); (b) length of stay in hospital and (c) health care costs associated with the therapy, transfer and ICU admission (including intubation rates), and overall length of stay; (d) length of oxygen therapy; (e) and measurement of adverse side effects. We hypothesise that HFNC therapy will have a lower failure proportion, reduced transfer rate, reduced length of stay, and lower health care costs associated with the treatment, in comparison to standard subnasal oxygen.

### Study design and settings

#### Design

Open label, non-blinded multi-centre, randomised controlled trial comparing respiratory support and oxygen delivery via HFNC therapy versus standard subnasal oxygen therapy in infants (corrected age) to 12 months of age admitted to hospital with bronchiolitis.

#### Setting

Emergency departments and general paediatric wards of 17 hospitals in Australia and New Zealand, including 12 non-tertiary regional/metropolitan and 5 tertiary centres. Eight hospitals will have access to on-site intensive care units that cater for infants less than 12 months of age. Patients will be recruited in both the ED and general paediatric wards of these hospitals.

#### Ethical consideration and delayed consent

The study has ethical approval for delayed consent for all except one of the participating centres, as Ethics within their State does not accept delayed consent under the Interstate Mutual Acceptance initiative (Children’s Health Queensland HREC/13/QRCH/93, New Zealand Health and Disability Ethics Committees HDEC/15/CEN/46). Informed consent will be sought from the parent or guardian of the infant by the Research Nurse, Study Coordinator, or relevant clinician as soon as possible once the infant has been stabilised and the parent/guardian has had time to adjust to the emergency environment. The parents/guardians can withdraw at any time during the study, the information already collected will be retained, to ensure the results of the study can be properly evaluated.

The trial is overseen by a steering committee responsible for the ethical and rigorous conduct of the trial in both Australia and New Zealand. Governance approval has been obtained from all study sites. A Data Safety and Adverse Event Monitoring Committee has been appointed with membership independent from the conduct of the trial.

#### Case selection

Infants meeting all inclusion and exclusion criteria will be identified at triage in all EDs. This includes identifying all patients <12 months of age with respiratory symptoms and an oxygen requirement (less than 92 % saturation for tertiary centres and less than 94 % saturation for metropolitan and regional centres). Patients who are admitted to the paediatric ward without an oxygen requirement on admission but later meet these inclusion criteria are to be recruited into the study from the ward environment.

### Definitions

All infants aged 0-12 months with symptoms of bronchiolitis (as defined by the American Academy of Pediatrics) - symptoms and signs of respiratory distress and oxygen requirements are eligible [18]. Bronchiolitis is defined as signs and symptoms of respiratory distress associated with symptoms of a viral respiratory tract infection (cough, runny nose, blocked nose, tachypnoea, recessions, nasal flaring, and cyanosis) [3]. Oxygen requirement in room air is defined according to the World Health Organisation recommendation, if transcutaneous haemoglobin oxygen saturation (SpO_2_) is <94 % in room air [19–21]. Six participating tertiary centres in our study have a lower threshold of < 92 % as their standard practice of care. In these sites a threshold of < 92 % was accepted in the randomised design, different saturation thresholds in different centres will be balanced through randomisation across all sites.

***Inclusion criteria*** are infants with a clinical diagnosis of bronchiolitis less than 12 months corrected age (≥ 37 weeks post-conceptional age) with an oxygen requirement in room air of SpO2 < 92 % for tertiary centres and <94 % for regional and metropolitan centres.

***Exclusion criteria*** include those infants with urgent need for respiratory support ( i.e. non-invasive or invasive ventilation or low level of consciousness, apnoea), cyanotic heart disease, basilar skull fracture, upper airway obstruction, craniofacial malformations and infants who are already on home oxygen therapy.

### Primary outcome definition

The primary outcome is the proportion of infants in each group with treatment failure. Treatment failure of either standard subnasal oxygen or HFNC therapy arm is defined as meeting three out of four specified failure criteria requiring escalation of treatment or higher level of care such as high acuity or intensive care. Prior to escalation of care 2 to 3 hours of observing the infant in an undisturbed environment is suggested. This time frame was based on the pilot study whereby it was clearly identified that the infants who are going to fail will do so within 2 to 3 hours [12]. In the pilot study, those who failed HFNC therapy had unchanged or increasing heart rate and respiratory rate, or worsening SpO2. Clinicians are asked to wait 2 to 3 hours prior to escalation of care, however, if they are more concerned, their clinical judgement can override the protocol if a decision is made to escalate care.

***Treatment failure*** of either standard subnasal oxygen or HFNC therapy arm is defined if three of the four following criteria are met and an escalation of treatment or level of care is required

- heart rate remains unchanged or increased compared to admission/enrolment observations,
- respiratory rate remains unchanged or increased compared to admission/enrolment observations,
- oxygen requirement in HFNC therapy arm exceeds FiO_2_ ≥ 40 % to maintain SpO_2_ ≥ 92 % (or ≥94 %) or oxygen requirement in standard subnasal oxygen therapy arm exceeds >2L/min to maintain SpO_2_ ≥ 92 % (or ≥94 %)
- hospital internal Early Warning Tool calls for medical review and escalation of care.

The study design utilises a ***composite primary end-point of treatment failure***, which has previously been used in a RCT of HFNC therapy vs. CPAP post extubation in neonates conducted in Australia [22].

### Secondary outcome definition

Secondary outcomes are defined as (a) the proportion of infants requiring transfer to higher acuity care, which includes admission to an on-site paediatric intensive care or transfer to a tertiary hospital; (b) length of hospital stay, including intensive care length of stay and (c) intubation rates; (e) associated health care costs for the respective therapy; (f) length of oxygen therapy and; (g) adverse events.

#### Randomisation process

The enrolment/randomisation process has been successfully tested in the previous pilot study. Randomisation will be achieved by an opaque envelope in sequential order with an allocated infant ratio of 1:1. A computer-based randomisation will be used to allocate infants 1:1 per site. Randomisation can occur at admission or at any time during hospital stay when an oxygen requirement develops.

### Treatment arms

#### Control

The infant that is randomised to the Control arm will be placed on standard subnasal cannula with oxygen flows up to a maximum of 2L/min according to local hospital practice to maintain SpO2 between 92-98 % (94-98 %). This may or may not be humidified oxygen, and will be according to current individual hospital guidelines. Oxygen flows should be administered below 2L/min to commence with and increased as required to maintain SpO2 ≥ 92 % (≥ 94 %).

#### HFNC therapy

The infant that is randomised to the HFNC therapy arm with SpO2 85-91 % (85-93 %) will be placed on high flow at 2L/kg/min (up to a maximum of 25 L/min) in room air equating to a fraction of inspired oxygen of 21 % (FiO2). They will remain on room air for a minimum of 10 minutes whilst monitoring SpO2. If SpO2 starts to increase and stabilise at ≥92 % (≥94 %) then continue on room air of 21 %. If SpO2 has not altered or improved then the FiO2 will be slowly increased to maintain SpO2 ≥ 92 % (≥94 %). The infant that is randomised to the HFNC therapy arm with SpO2 < 85 % will commence on high flow with FiO2 slowly increasing to achieve SpO2 ≥ 92 % (≥94 %).

#### Other interventions/therapies/nutrition

The use of any medications such as steroids, antibiotics, antipyretics, bronchodilators and hypertonic saline is at the discretion of the attending clinicians and not prescribed in the protocol. These interventions will be recorded in the clinical research form (CRF). Feeding in standard subnasal cannula oxygen therapy is not prescribed and clinicians can choose to administer continuous or bolus feeds or use intravenous fluids. However, in the HFNC therapy intervention arm a nasogastric tube will be inserted, the stomach must be vented at 4 hour intervals, and no oral feeding will be allowed unless in stable weaning phase. Feeding rate should be prescribed as per individual hospital policy using nasogastric (bolus or continuous feeds).

The nursing ratio for both arms will remain according to the standard current ward management already present and as per usual hospital protocol. The nurse/patient ratio is important to maintain, to ensure that infants with the same level of illness do not get increased nursing care due to being on one type of therapy over another. Clinical documentation will be achieved according to individual hospital standards. Admission criteria to intensive care and triggering of medical review (use of early warning tool) will also follow individual hospital rules.

#### Choice of 2L/kg/min flow rate

Studies have demonstrated that the maximal inspiratory flow that a healthy infant achieves during regular breathing is 0.8 L/kg/min for each breath. An unwell infant such as an infant with bronchiolitis, generates a higher inspiratory flow which can be as high as 1.0-1.6 L/kg/min [10, 23]. The aim of HFNC therapy is to match this maximal inspiratory flow generated by the infant, so that no additional air is entrained around the nasal prongs. For this purpose a safety margin of 2 L/kg/min is given, to ensure adequate flow is provided for these infants.

There is a population of larger babies less than 12 months of age and the HFNC therapy for this study will not exceed 25L/min. This is based on using the infant’s ideal weight rather than actual weight as a measure of how to deliver the flow for the larger babies.

#### Weaning

Responders in the *standard subnasal oxygen therapy arm* will be weaned from 2 L/min or less if SpO2 remains stable at 92-98 % (94-98 %). Infants will be weaned to room air and nasal cannula removed once an infant is able to maintain saturations in the target range on room air for at least four hours. SpO2 to be maintained within 92-98 % (94-98 %) at all times and if <92 % (<94 %), then oxygen flow will increase again until SpO2 within 92-98 % (94-98 %). Once SpO2 is stabilised, weaning will recommence, using the same parameters.

For responders in the *HFNC therapy arm*, flow rates will be kept at 2L/kg/min, while FiO2 is decreased. Flow rates are *not* to be weaned and are to be maintained at 2L/kg/min until HFNC therapy is ceased and turned off completely. Once FiO2 has reached 21 % with SpO2 maintained between 92-98 % (94-98 %) for four hours the high flow off is turned off and the nasal cannula are removed.

#### Escalation of therapy after treatment failure (primary outcome)

Once failure criteria are met, escalation of treatment may be from standard subnasal oxygen therapy to HFNC therapy and/or escalation to non-invasive ventilation or intubation and mechanical ventilation in intensive care. It is at the discretion of the attending physician to manage and treat the ongoing care. If escalation from standard subnasal oxygen to HFNC therapy occurs then the same protocol shall be applied as in HFNC therapy arm. However, at any time during the study the physician can override and escalate care. This aspect of clinical reasoning to escalate will be captured in the data set and is defined as follows: i.) increased work of breathing requiring escalation of respiratory support, ii.) decreased conscious level, iii.) deterioration of cardiovascular function with impaired peripheral perfusion, iv.) clinical judgment of attending (senior) medical officer triggers escalation of treatment and care.

With any escalation of treatment the nurse/patient ratio will be captured to perform cost assessments on required level of care at the time of escalation and thereafter.

#### Clinical observations

SpO2 will be measured at enrolment in room air. If the infant has had oxygen administered in the ambulance, then it will be ceased and SpO2 assessed a few minutes later to ascertain if the infant meets the inclusion criteria of SpO2 < 92 % (<94 %). Once this inclusion criteria has been established, respiratory rate, respiratory effort and heart rate will be measured at enrolment. Observations thereafter will be according to current hospital protocol within either the ED or general paediatric ward.

At each set of observations, the following parameters will be documented: SpO2, heart rate, respiratory rate, respiratory effort, oxygen/FiO2 administered, flow rate of high flow therapy and at frequent intervals the temperature and blood pressure will be monitored according to standard hospital guidelines.

Complications recorded will include episodes of apnoea and bradycardia (lasting more than 20 seconds); all episodes where the SpO2 was recorded <92 % (94 %) and adverse outcomes such as pneumothoraces or deaths.

#### Laboratory investigations

Laboratory investigations are not mandated for the trial. However it is recommended that all enrolled patients have screening for respiratory viruses using nasopharyngeal aspirate completed to provide data on the various viruses amongst the recruited patients. The screening will occur at the discretion of the managing physician.

### Time frame

It is predicted that patient enrolment will occur over a period of three years. Individual participant study involvement will cease at death or discharge home.

### Healthcare cost analysis

A within-trial economic evaluation will be undertaken from the health care provider perspective using a cost-minimisation approach, to compare the cost of providing HFNC therapy to that of standard subnasal oxygen therapy. This approach assumes a reasonable and equivalent clinical outcome at the point of discharge to home. Resources to be measured and costed will include the number of transfers between hospitals, length of stay (in PICU and/or non-intensive stay), and resources related to use of the intervention (HFNC therapy or standard subnasal oxygen therapy). Costs will be assigned using standard costing sources [24]. The Australian refined diagnostic-related groups (AR-DRG) will be used to indicate the costs associated with each hospital admission, adjusted for length of stay in hospital and in PICU. The New Zealand centres health care costs will be analysed separately and sensitivity analysis for the different health care system will be performed. Parametric (e.g. ANCOVA) and non-parametric bootstrapping techniques will be employed to compare the costs between groups, and to estimate a confidence interval around the mean difference [25]. It is anticipated that HFNC therapy will be cost saving to the health system. The cost comparison will inform recommendations on adopting HFNC therapy for the management of bronchiolitis in clinical practice.

### Sample size, power and statistical methods

#### Sample size

In 2011 there were 478 eligible patients admitted to 4 of 8 pilot hospitals (Queensland), of which 80 required transfer to a tertiary paediatric centre (16.7 %). Assuming a conservative baseline rate of failure of standard therapy (need for transfer to a specialist paediatric centre or PICU admission) of 10 %, a 50 % relative reduction to 5 % with a power of 90 % and type I error of 0.05, 582 participants per group are required, resulting in a total sample size of 1164 patients. An attrition rate of approximately 10-20 % is estimated, which gives an overall sample size of 1400.

#### Data analysis

Descriptive statistics will be utilised to report on the baseline characteristics of the total study cohort, as well as by site. The primary outcome measure investigating clinical treatment failure will be analysed using test of two proportions, and will be reported as the difference, 95 % confidence interval and *p*-value. The secondary outcome measure investigating length of stay will likely be non-normally distributed, and as such will be analysed using a Mann-Whitney U test; if it is normally distributed independent samples t-test will be used. Analysis of secondary outcomes includes both comparisons of measurements and proportions, using confidence intervals of differences as the major method of presentation where possible. A logistic regression analysis will be used to identify risk factors. All analyses will be by intention-to-treat. A per protocol analysis will be undertaken as a secondary analysis. Pre-specified sub-groups include; ex-preterm infants, infants with congenital heart defect, infants less than 3 months of age (corrected), infants less than 6 months of age (corrected), infants presenting to tertiary sites, infants presenting to secondary sites. Statistical significance will be set at the 0.05 level. No interim analysis will occur and no stopping rules were defined.

### Adverse events and monitoring/reporting

#### Data safety monitoring

An external data safety monitoring board has been established. All adverse events and experiences by either staff or parent/guardian will be reported on the CRF provided for each patient and the local Principal Investigator notified who will in turn notify the Chief Investigator. A serious adverse event (SAE) is defined as any event that is fatal, life-threatening, permanently disabling, incapacitation or prolongs a hospital stay.

#### Reporting SAE

SAEs will be reported within 24 hrs by telephone to the local Principal Investigator who in turn will notify the Chief Investigator. The local site investigator will report the SAE to their local ethics committee within 48 hours (or in accordance with local ethics committee regulations).

#### Limitations

As a non-blinded trial there is the potential for the investigator to unintentionally introduce measurement or reporting bias because further knowledge of the study and treatment allocation could influence the clinicians’ decision-making. The end points can be seen as subjective and we cannot alter the medical and nursing staff’s opinion at certain points in the trial. As an example, some patients may be seen as not responding to enrolled arm treatment at one centre or on one shift whereas another centre or the next shift of staff may view this differently. It is not possible in this trial to blind the investigators or treating physicians.

#### Current status of trial

The study enrolment has commenced, and are expecting all 17 sites to be recruiting by end of July 2015.

***Sites involved in the study include:***

***Australia:***

*Queensland:*

Ipswich Hospital, Gold Coast University Hospital, Caboolture Hospital, Nambour Hospital, Logan Hospital, Redland Hospital, Redcliffe Hospital, The Prince Charles Hospital, Toowoomba Hospital, Townsville Hospital, Lady Cilento Children’s Hospital

*New South Wales:*

The Tweed Hospital

*Victoria:*

Royal Children’s Hospital Melbourne

Monash Health

*ACT:*

Canberra Hospital

***New Zealand:***

Starship Children’s Health

KidzFirst, Middlemore Hospital

## Discussion

This large multicentre randomised trial will allow the definitive assessment of the efficacy of high flow nasal cannula therapy as compared to standard subnasal oxygen in preventing treatment failure and the need for escalation of respiratory support. This study will in addition provide a unique insight into the adverse events profile of high flow nasal cannula therapy when used outside the intensive care setting and the epidemiology of the moderate to severe end of the spectrum of bronchiolitis.

### Time plan

To date (July 2015), 380 patients have been recruited for the planned 1400 patients with recruitment to be completed in 2017.
